# A Complete Key Management Scheme for LoRaWAN v1.1 †

**DOI:** 10.3390/s21092962

**Published:** 2021-04-23

**Authors:** Xingda Chen, Margaret Lech, Liuping Wang

**Affiliations:** School of Engineering, RMIT University, Melbourne, VIC 3000, Australia; margaret.lech@rmit.edu.au (M.L.); liuping.wang@rmit.edu.au (L.W.)

**Keywords:** IoT, security, LoRaWAN, LPWAN, key management, key generation

## Abstract

Security is one of the major concerns of the Internet of Things (IoT) wireless technologies. LoRaWAN is one of the emerging Low Power Wide Area Networks being developed for IoT applications. The latest LoRaWAN release v.1.1 has provided a security framework that includes data confidentiality protection, data integrity check, device authentication and key management. However, its key management part is only ambiguously defined. In this paper, a complete key management scheme is proposed for LoRaWAN. The scheme addresses key updating, key generation, key backup, and key backward compatibility. The proposed scheme was shown not only to enhance the current LoRaWAN standard, but also to meet the primary design consideration of LoRaWAN, i.e., low power consumption.

## 1. Introduction

The Internet of Things (IoT) has experienced rapid growth in recent years. According to [1] the total number of inter-connected devices in the world will reach more than 50 billion in 2023 and it is expected to exceed 100 billion devices by 2025 [2]. Moreover, the total IoT revenue is expected to reach US $19 trillion United States dollars by 2025 [2]. The majority of different connections between IoT devices are wireless. The Low Power Wide Area Network (LPWAN) is a class of wireless access technologies that are characterized by low power consumption and long-distance coverage. In certain applications, LPWANs have their own advantages over short-range wireless technologies such as Wireless Fidelity technology (Wi-Fi) and Bluetooth because short-range wireless communication protocols have the advantage of high data rates but it usually comes with high energy consumption. Unfortunately, most IoT devices such as Radio-Frequency Identification devices (RFIDs) or smart cards, have limited resources such as limited power and low computing and processing abilities [3,4]. LoRaWAN stands for a Low Power, Long Range Wide Area Network, it is a Medium Access Control (MAC) layer networking protocol. It has been designed to connect mobile or stationary power-constrained devices via two-ways communication wirelessly in multiple domains such as national or regional. LoRaWAN is one of the most popular LPWAN standards quickly adopted in IoT applications. The paper [5] indicates that 70% of the IoT devices are highly vulnerable to security attacks. Given the observed steady growth of IoT devices, IoT security becomes one of the key factors ensuring internet reliability. Therefore, the design of efficient IoT security systems is of high importance. One of the ways to defend the LoRaWAN security is to have a strong key management security framework [6,7,8,9,10,11,12,13,14,15]. The current key management security framework is provided in the LoRaWAN v1.1.

The LoRaWAN v1.1 was released on 11 October 2017 [16], offering a significant improvement of the key management security aspect. However, LoRaWAN v1.1 still has some limitations and drawbacks in its key management scheme, which can pose danger to its security defense. [6,17,18] have addressed the importance of using a new session key generation algorithm rather than the traditional algorithm used by the current release due to the traditional algorithm is considered computational heavy for the IoT end devices. IoT end devices’ security will be compromised if the traditional algorithm is used, since the limitation of secret key updates from the constrained battery power, which would let the attackers have enough time to decipher the secret key [19,20]. Also, the current key updating scheme is not clearly defined [7]. Key back-up, recovery, revocation, and handling of backward compatibility issues [21,22,23,24] are existed in the current version. Those issues make the key management security framework vulnerable. Currently, there are a few key management schemes that existed in the literature [7,8,9,10,11,12]. However, each focused on a different aspect of the key management, more details can be found in Table 1 of Section 1. For example, in the paper [8], they focused on the static root key updates, our approach focused on the session key generation and believed that the more frequent session key updates with the support of the key updating scheme can reduce the chance of exposing the session keys even the root keys. Hence, the need for root key updates is reduced. Also, most key management schemes covered only one aspect of the security key management, but our paper has addressed more areas such as providing guidelines to handle session key update, key back-up, recovery, revocation, and handling of backward compatibility issues.

Hence, our contribution to the field is that, at first, we did a thorough security structure analysis of LoRaWAN v1.1 based on the American National Institute of Standards and Technology (NIST) key management guideline [25], to conduct analysis for the secret key lifecycle which is a novel approach based on our best knowledge and listed out the potential vulnerabilities and limitations of the key management scheme. After that, we provide a complete guideline to tackle ambiguously defined issues that exist in the LoRaWAN v1.1 key management scheme, such as the frequency of the session key update, key back-up, recovery, revocation, and handling of backward compatibility issues. At the last, most importantly, a key generation algorithm based on the Rabbit Pseudo-Random Number Generator (PRNG) is proposed to enhance the overall speed of the key generation.

The remaining part of this paper is structured as follows. In Section 2, the most recent literature reviews are conducted, strengths and weaknesses of different approaches are analyzed. In Section 3, the structure of the LoRaWAN is discussed, how does the LoRaWAN work is explained. In Section 4, the security analysis of LoRaWAN v1.1 is presented, including the key management of LoRaWAN and the drawbacks of LoRaWAN v1.1. In Section 5, a complete key management scheme of the LoRaWAN for the Over the Air Activation (OTAA) devices is proposed and validated. In Section 6, a proposed Pseudo-Random Number Generator (PRNG) for the session key derivation is designed, implemented, evaluated, and the results of the proposed PRNG with other session key generators are compared. In Section 7, a discussion about our proposed solutions and how our contributions are made to the field are discussed. In Section 8, a conclusion is drawn.

## 2. Related Work

To address the above security vulnerabilities, different authors have proposed different key management solutions for LoRaWAN in recent works. Strengths and weaknesses of the most recent works have been examined.

Han and Wang [8] reviewed LoRaWAN v1.1 security framework and enhanced a root key update scheme that makes the LoRaWAN root keys more secure, because static root keys throughout the lifetimes of the device can make attacker has enough time to obtain knowledge of the session key derivation. The experimental results show that this scheme generates a high degree of randomness with higher computing efficiency compared to hash-based Key Derivation Function (KDF). However, the paper [8] did not have other solutions to address the session key update issue, key back-up, recovery, revocation, and backward compatibility issues. Hence, that makes our paper a more complete key management scheme. Furthermore, the randomness test in [8] was using 1000 key samples from the outputs of the Rabbit-based KDF which are used to calculate the standard deviation and Probability Density Function (PDF). The output has shown evenly spread block values. On the other hand, our randomness tests were strictly based on the NIST Randomness Guidelines [26]. Hence, a strict randomness performance can be ensured.

You et al. [9] evaluated LoRaWAN’s security vulnerabilities and presented a key exchange protocol based on the enhanced Elliptic Curve Diffie Hellman (ECDH), the results have shown that better performance (network latency and signal overhead reduction) for the proposed protocol in comparison with other security protocols, namely Datagram Transport Layer Security-Pre-Shared Key (DTLS-PSK) and Datagram Transport Layer Security-Elliptic Curve Cryptography (DTLS-ECC). Although the paper [9] focused on the v1.02 of the LoRaWAN framework, the paper [9] has indicated compatibles with version 1.1 of LoRaWAN. The reason is that they have proposed a protocol to allow the end device and the application server to exchange the session key securely by using the ECDH-based key exchange and the Elliptic Curve Digital Signature Algorithm (ECDSA) digital signature to overcome the problem that the network server has known the AppSKey, which aims to protect the application data.

Sanchez-Iborra et al. [7] reviewed LoRaWAN’s security vulnerabilities and suggested a different approach based on the Ephemeral Diffie-Hellman Over COSE (EDHOC), where COSE stands for Concise binary object representation Object Signing and Encryption, due to its flexibility in the update of session keys, its low computational cost, and the limited message exchanges needed. Their contribution is to reduce the handshake overhead by about 31% than using the Datagram Transport Layer Security (DTLS) Protocol. Similar to You et al. [9], both solutions focused on key exchange protocol. Therefore, some works could be done on other areas to further improve the LoRaWAN key management scheme. For example, the session key generation, by using a more efficient session key generator can further reduce the cost of the key update, since more keys can be produced to allow more frequent key updates.

Xing et al. [10] presented a secure key management scheme for static key update remotely using a Hierarchical Deterministic (HD) wallet and key exchange through an Elliptic Curve Diffie-Hellman (ECDH) algorithm. The result shows that the key exchange algorithm performed better than Diffie-Hellman (DH) algorithm. Similar to [8], both focused on key updating of the static root keys. Also, similar to [7,9], the key exchange algorithms have been improved.

Dönmez and Nigussie [11] in the paper stated a key management scheme by using a delegation named Assisted Mode to overcome the security concern of the static root keys due to the lifetime of the static root keys can expose the weakness to attackers. Unlike [8,10] require algorithms to remotely update root keys. The proposed scheme delegates the management of the root keys to a master device (a device with high computing power and more resilient to attacks). Thus, the end devices are no longer under security threat for exposing the root keys. However, this scheme heavily relies on the assumption that the master device and end-devices must be closed to each other. Hence, this scheme might not be applicable if the master device is not able to allocate close to the end devices.

Ribeiro et al. [12] are proposed a secure architecture for key management based on smart contracts and permissioned blockchain to enhance security and availability in LoRaWAN networks. The blockchain allows multiple peers (similar roles to the join server) instead of one join server to ensure the availability of the handling of the OTAA procedure and storing copies of all encryption keys. Also, all OTAA attempts would be kept by all peers allows clear auditing. No further changes are needed to the framework due to all changes are related to the join-server. However, due to all changes are done on the join server-side, it overcomes the problem related to the failure of the join server, but the issues associated with other parts of the network might still exist. For example, the malicious attacker still has the chance to decipher the application data if the attacker can crack the session key from the result of infrequent key updates. Hence, inefficient session key generations and infrequent key updates are still posed security challenges.

Furthermore, we reported a fast session key generation algorithm to handle the session key generation part of the key management in [6]. However, this paper presents further detailed security analysis with a complete key management scheme discussed below. Also, a higher efficient session key generation algorithm is developed since then. In this paper, the Modified Rabbit v1 refers to the key generation algorithm in [6]. Our enhanced session key generation is 14.8% faster than the algorithm in [6]. A detailed discussion of the work is conducted in Section 6 of this paper.

In contrast to the works just described, our work covered the gaps that some of the abovementioned schemes did not cover. A detailed discussion of our works and other key management schemes can be found in Section 7.

## 3. LoRaWAN v1.1 Structure Analysis

**Figure 1:**
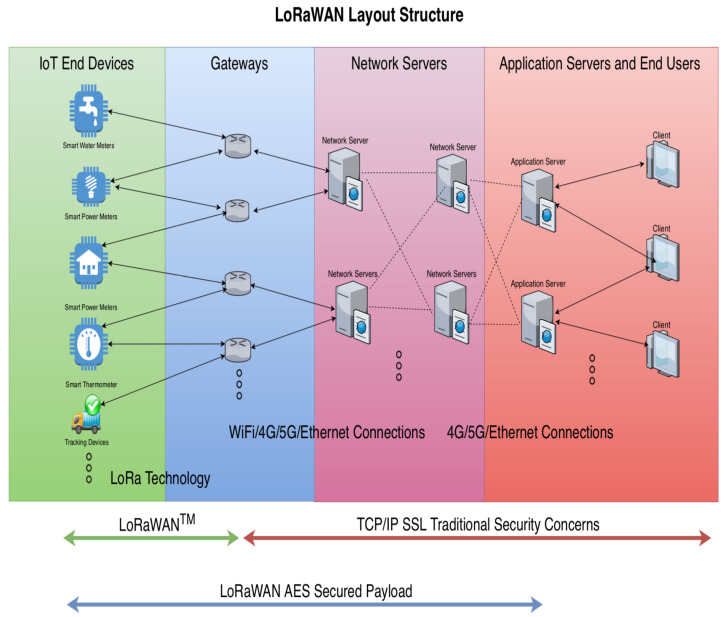
The layout of the LoRaWAN structure.

In general, the LoRaWAN network is comprised of the following elements: end devices (typically a constrained devices), gateways (purely relay messages, or calls base stations), network servers, application servers, and join-servers. A LoRaWAN network has a star of stars topology. The gateways relay the data between end-devices and a network server, a network server directs the data between end devices and the paired application server. A join server comes to play at the initial stage when the device joins/rejoins the network. The join server generates and distributes the pairs of session keys from their devices’ root keys to each player in the network (i.e., the corresponding network server and the application server). The device would successfully establish a secured radio channel using the Advanced Encryption Standard (AES 128) symmetric block cipher once it joins the network. Figure 1 illustrates the layout of the LoRaWAN structure.

Furthermore, the end-devices collect service-oriented data from the environment and send it to the gateway via a single hop LoRa technology (an LPWA technology). Then the data is sent to the correct network server and vice versa. A mobile end device can receive data that traveled through one or many gateways before reaching the correct network server. Gateways are connected to the network server through a secured standard Internet Protocol (I.P.) connection. All communications are generally bi-directional, although the uplink communication from an end-device to the network server is expected to generate the majority of the network traffic. Communication between end devices and gateways are spread out across different frequency channels and data rates. It uses the LoRa, a radio modulation technology that is based on Chirp Spread Spectrum (CSS) running on Industrial, Scientific and Medical (ISM) unlicensed bands. The frequency bands differ from regions that apply EU868, EU433, US915 and AS430 standards [8]. To maximize both the battery life of the end-devices and the overall network capacity, the LoRa network infrastructure manages the data rate and radio-frequency output for each end-device individually via an Adaptive Data Rate (ADR) scheme. The network server is the center of the star of stars topology. This is where the Medium Access Control (MAC) payload is deciphered, and the cipher application message is transmitted to the application server. The application server processes the application-layer payloads.

Depending on the end-devices’ functionality, there are three different classes of LoRaWAN: Class A, Class B and Class C for the end-devices. Class A allows bi-directional communications with each end-devices with one uplink transmission that follows two short downlinks receive windows. Class A has the lowest power consumption, and all LoRaWAN devices must have the capability of implementing it. Class B allows for more receive windows at scheduled times. These times are synchronized with the gateway beacon. Class C allows continuously opening the receiving windows unless transmitting. Hence, it has the highest power consumption and the lowest service latency.

In the LoRaWAN Join Procedures, an end-device has two ways of joining the LoRaWAN network. (1) using the Over the Air Activation (OTAA) procedure. (2) using the Activation by Personalization (ABP) procedure. During the OTAA procedure, two messages are being exchanged (Join-Request and Join-Accept message) between the end device and network server, four session-keys (FNwkSIntKey, SNwkSIntKey, NwkSEncKey and AppSKey) are being generated after receiving the accept message. In the ABP procedure, on the other hand, the four session keys are pre-installed on the LoRa device, so that the device is ready to participate in a specific network at any time.

**Figure 2:**
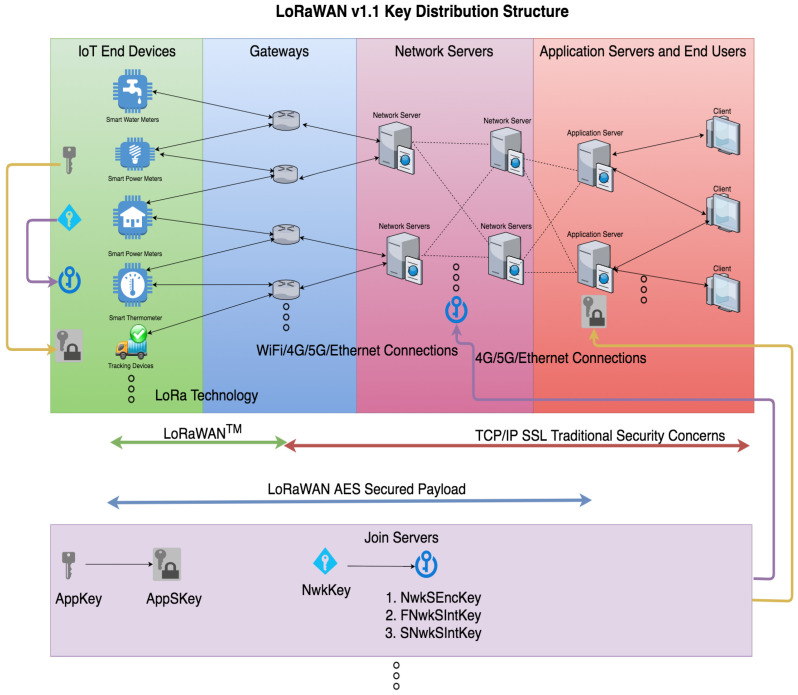
LoRaWAN v1.1 Key Distribution Structure.

Figure 2 illustrates the LoRaWAN v1.1 key distribution structure. Compared with the earlier LoRaWAN v1.0 released in January 2015 [16], the most significant change is the additional device root-key. In the 1.0 version, there is only one device root-key. However, there are two device root keys in the LoRaWAN v1.1: A Network Key (NwkKey) and an Application Key (AppKey). While the Network Key (NwkKey) in the 1.1 version is equivalent to the Application Key (AppKey) in the 1.0 version, the extra key (AppKey) in the 1.1 version is to ensure that the network operator would not be able to see the application data. Hence, the confidentiality of the message is secured. In addition, to strengthen the security, the four session-keys (FNwkSIntKey, SNwkSIntKey, NwkSEncKey, and AppSKey) are derived from the two device root-keys. The first two Network session keys (FNwkSIntKey and SNwkSIntKey) are used to ensure the data integrity for both uplink and downlink. The Network Session Encryption Key (NwkSEncKey) is used to encrypt and decrypt the uplink and downlink MAC payloads. The Application Session Key (AppSKey) is used to encrypt and decrypt the application payloads. Thus, the confidentiality of the data is secured.

## 4. LoRaWAN v1.1 Security Structure Analysis

According to the National Institute of Standards and Technology (NIST)’s recommended key management guideline [25], the secret key lifecycle consists of the following four phases: (1) pre-operational phase, (2) operational phase, (3) post-operational phase, and (4) destroying phase. The key generation and distribution processes are parts of the pre-operational phase. Key storage, usage, backup, and recovery are parts of the operational phase. Key expiry and updating are taking place in the post-operational phase. Finally, the key revocation takes place in the destroying phase. To analyze LoRaWAN v1.1, the following sub-sections examine each stage of the key management lifecycle.

### 4.1. Pre-Operational Stage

The generation and distribution of the secret keys of LoRaWAN v1.1 are closely related to its network join procedure. Before joining the network, the following items are pre-installed in the LoRa devices.

- For OTAA devices: JoinEUI (it is a global unique join server identifier (I.D.) in Institute of Electrical and Electronics Engineers (IEEE) Extended Unique Identifier (EUI) 64 bits address space). DevEUI (it is a global unique end device I.D. in IEEE EUI 64 bits address space). Device’s 128 bits AES root keys AppKey and NwkKey (both keys are essential to derive the group session keys).
- For ABP devices: No join procedure is required due to the device with pre-stored the four session keys (FNwkSIntKey, SNwkSIntKey, NwkSEncKey and AppSKey). Hence, the key generation and distribution procedures are not needed.

In addition, in the OTAA case, the LoRa device sends a Join-Request message to the network server, and then the join server handles the message.

As shown in Table 2, the Join-Request message contains the 1 byte MAC Header (MHDR), 8 bytes JoinEUI, and 8 bytes DevEUI of the end device information followed by a 2 bytes Device Nonce (DevNonce) and a 4 bytes Message Integrity Code (MIC). The DevNonce is to ensure that the message is free from replay attacks. Since the DevNonce is a counter starting from zero and counting up at each Join-Request, it cannot be reused for the same JoinEUI. Hence, the network server can keep track of the last DevNonce value used by the end-device, and ignores the Join-Request message, if DevNonce is incorrect. Although the Join-Request message is not encrypted [16], the data integrity can be ensured by using the Message Integrity Code (MIC) value. The MIC is calculated in (1) as the first 4 bytes of the Cipher-based Message Authentication Code (CMAC) code [16],

(1) MIC=cmac[0..3]

where the CMAC code is given as,

(2) cmac=aes128_cmac(NwkKey,MHDR|JoinEUI|DevEUI|DevNonce)

the aes128_cmac in (2) represents an authentication algorithm based on Cipher-based Message Authentication Code (CMAC) with the 128 bits AES.

The network server confirms with the join server once it receives the Join-Request message. To check if the end-device is authentic, the join server examines the DevEUI and MIC to see if they are matched. Once the device is determined to be legitimate, the join server sends a JoinNonce. The JoinNonce is a device-specific counter value incremented with every Join-Accept message, so the value never repeats itself. It can be used to prevent replay-attacks on the paired network server. After the JoinNonce, the network server sends a Join-Accept message. As shown in Table 3, the Join-Accept message consists of a 1 byte MHDR, a 3 bytes JoinNonce, a 3 bytes network identifier (NetID), a 4 bytes end device address (DevAddr), a 1 byte DLSettings field (providing downlink settings), a 1 byte delay time between transmit and receive window (RxDelay) and finally, a 16 bytes optional list of network parameters (Channel Frequencies) that the end device is joining (CFList), as well as a 4 bytes MIC at the end of the message.

The end device is able to derive the network session keys (FNwkSIntKey, SNwkSIntKey, and NwkSEncKey) and the application session key (AppSKey) once the JoinNonce is received. The root keys (NwkKey and AppKey) are only stored in two places, the end-device, and the join server. The root keys are only valid for the lifetime of the end device. Moreover, the root keys are never be sent or received over the air. The network session keys have different roles: (1) The Network Session Encryption Key (NwkSEncKey) is responsible for the MAC payload encryption between the end-device and the network server; (2) The Forwarding Network Session Integrity Key (FNwkSIntKey) is in charge of the message integrity check for uplink; (3) The Serving Network Session Integrity Key (SNwkSIntKey) is the message integrity check for downlink; The Application Session Key (AppSKey) is taking care of the application payload encryption/decryption between end-device and application server. The four network session keys can be derived as follows [16].

(3) $$NwkSEncKey=aes_{128}encrypt(NwkKey,0\times 04|JoinNonce|JoinEUI|DevNonce|pad_{16})$$

where aes_128 encrypt in (3) represents 128 bits AES encryption function using Electronic Codebook (ECB) mode.

(4) $$FNwkSIntKey=aes_{128}encrypt(NwkKey,0\times 01|JoinNonce|JoinEUI|DevNonce|pad_{16})$$

(5) $$SNwkSIntKey=aes_{128}encrypt(NwkKey,0\times 03|JoinNonce|JoinEUI|DevNonce|pad_{16})$$

(6) $$AppSKey=aes_{128}encrypt(AppKey,0\times 02|JoinNonce|JoinEUI|DevNonce|pad_{16})$$

Equations (3)–(6) indicate that there are three parameters JoinNonce, JoinEUI, and DevNonce that are essential to the key derivation. Also, JoinEUI and DevNonce are not encrypted during the join procedure, meaning that these parameters are known to the public.

### 4.2. Operational Stage

#### 4.2.1. Key Usage

The LoRaWAN v1.1 supports the backward capacity, which makes LoRaWAN particularly vulnerable when the network operator is acting as the join server. It can happen when the join server is absent. The operator, in this case, has the knowledge of the NwkKey, which enables him to decipher the network session key and the application session key in v1.0 [16]. If the join server is absent, the network server only supports the version below LoRaWAN v1.1, the AppSKey derivation given as [16],

(7) $$AppSKey=aes_{128}encrypt(NwkKey,0\times 02|JoinNonce|JoinEUI|DevNonce|pad_{16})$$

Therefore, the latest LoRaWAN protocol must be implemented to ensure the system that has maximum security.

##### (a) Confidentiality

The content of the data transmission is covered by the payload encryption. The MAC frame payload is encrypted using AES 128. The keystream(S) generation and the payload encryption/ decryption are detailed as follows [16]. For each data message, a sequence of Ai blocks is defined by the algorithm for i = 1,...,k, as,

(8) Ai=(0×001|0×004|Dir|DevAddr|FCntUporNFCntDwnorAFCntDwn|0×00|i)

where Dir in (8) is the direction field with 0 value indicating uplink frames and 1 for downlink frames. The k value is determined as,

(9) k=ceil(len(pld)/16)

where

(10) pld=FRMPayload.

The Frame Counter UP (FCntUp) is the counter for uplink from the device to the network server, and the Network Frame Counter Down (NFCntDown) is the counter for the downlink from the network server to the device. The Application Frame Counter Down (AFCntDown) is used for all other ports. Then, a sequence S=S1 |S2 |.. |Sk of Si(i = 1..k) blocks is produced from the encrypted blocks Ai,

(11) $$Si=aes_{128}encrypt\left(K,Ai\right)$$

where the key K is either NwkSEncKey or AppSKey depending on Port Field (FPort) setting. After that, the payload encryption (or decryption) is performed by truncating the sequence S xor (exclusive or) with the (pld | pad16), where pld is the frame payload.

##### (b) Message Integrity

The Message Integrity Code (MIC) is calculated to ensure data integrity by preventing the replay and bit-flipping attacks. It is checked by the network server and the end-device to make sure that the message is not altered by any third party. If the MIC value is incorrect, the relevant party discards the message.

- For Downlink Message [16]:

(12) $$cmac=aes_{128}\_cmac\left(SNwkSIntKey,B_{0}|msg\right),MIC=cmac\left[0..3\right]$$

where $aes_{128}\_cmac\left(\right)$ in (12) is an authentication algorithm using Cipher-based Message Authentication Code (CMAC) with the 128-bit Advanced Encryption Standard (AES).

(13) $$B_{0}=\left(0\times 49,ConfFCnt,2\times 0\times 00,Dir=0\times 01,DevAddr,AFCntDwn\;or\;NFCntDwn,0\times 00,len\left(msg\right)\right)$$

Confirmed Frame Counter (ConfFCnt) is the counter value modulo $2^{16}$ of the confirmed uplink frame, apart from that the ConfFCnt = 0x0000.

- For Uplink Message [16]:

(14) $$B_{0}=\left(0\times 49,0\times 0000,2\times 0\times 00,Dir=0\times 00,DevAddr,FCntUp,0\times 00,len\left(msg\right)\right),\;$$

(15) $$B_{1}=\left(0\times 49,ConfFCnt,T\times Dr,T\times Ch,Dir=0\times 00,DevAddr,FCntUp,0\times 00,len\left(msg\right)\right)$$

where TxDr is the uplink data rate, TxCh is the index of the transmission channel.

(16) $$cmacS=aes_{128}\_cmac\left(SNwkSIntKey,B_{1}|msg\right)$$

(17) $$cmacF=aes_{128}\_cmac\left(FNwkSIntKey,B_{0}|msg\right)$$

For LoRaWAN v1.0 network server,

(18) MIC=cmacF[0..3]

For LoRaWAN v1.1 network server,

(19) MIC=cmacS[0..1]|cmacF[0..1]

#### 4.2.2. Key Storage

The storage of root keys NwkKey and AppKey on the end device and the join server are the most vital parts of the overall security of LoRaWAN. However, LoRaWAN v1.1 does not describe in detail how the key should be stored. It only suggests that the implementation of it can include Secure Elements (SE) and Hardware Security Modules (HSM). Hence, the root keys must be stored in a way that the unauthorized party cannot gain access to it even by the side channel analysis.

#### 4.2.3. Key Backup and Recovery

This part is not discussed in the LoRaWAN v1.1. In the case of join sever accidentally deletes the end device root keys, it must have a backup server that can make the root keys recoverable. Session key backup and recovery are not an issue because the end device or the relevant servers can always ask the end device to re-join the network. However, the smoothness of the key backup and recovery is of concern since an improper transition may result in an unpleasant quality of service.

### 4.3. The Post-Operational Stage

#### Key Expiry and Updating

Key expiry and updating schemes are not fully specified in the LoRaWAN v1.1. Since all the secret keys are essential to protect the confidentiality of the message, it must be stored in a secret way. However, attackers keep finding these keys through the network traffic analysis, side channel analysis and other methods. Hence, the key updating is an effective way of avoiding the key exploration by attackers. Periodical updating is essential, also in the event of unusual behavior happening in the network. Hence, a secure key updating policy is paramount to the network security.

### 4.4. The Destroy Stage

This stage is not fully discussed in the protocol since the root keys are never to be updated in the current protocol, so there are no root keys to destroy at this stage. The session keys are only being replaced by new keys when the new session keys come into effect. It creates a risk of tracing back the old keys. Therefore, a specific policy for the old keys destroying stage should be implemented in the future LoRaWAN versions.

### 4.5. The Potential Security Vulnerabilities of LoRaWAN v1.1 Key Management Scheme

LoRaWAN v1.1 has presented the key derivation and distribution scheme and has ensured both confidentially of the network and application payloads as well as the integrity of the payload messages, the secret root keys are used for authentication of the end device. In this section, vulnerabilities and potential security risks of LoRaWAN v1.1 key management scheme are discussed.

In the LoRaWAN v1.1 specification, the key generation scheme is vague and outlines only general security requirements. A secure and efficient key generator procedure is essential to ensure a secured system that is hard to breakthrough. Since secret keys are essential to the overall security of LoRaWAN, key independence ensuring that one key procedure does not infer, another key must be implemented. Thus, a high level of randomness in the key generation is needed. Low randomness in the key generation poses a danger that some traits of one key can enable a cryptanalytic deduction of other keys [27]. Moreover, a key generator needs to be computationally efficient. The current version of LoRaWAN includes the AES algorithm, which is used for encryption and decryption. Hence, the current key generator appears to be computationally heavy for an efficient key generation. A solution improving both the key randomness and computational efficiency is proposed in Section 6 of this paper.

LoRaWAN has introduced new updating methods for session keys by using the ReJoin-Request message, which is always initiated by the end device. However, the recommended frequency of the session key updating scheme is not specified in detail. Therefore, questions like how often and under what circumstances should the key be updated or under what situation should the key be updated need to be answered. A high key-updating frequency at a low computational cost would lead to an increased network security [28]. In addition, the high frequency of key updating should not compromise the energy cost of the device too much. Otherwise, it would affect the lifetime of the device deployment.

As join servers hold the root keys of the end devices. The root keys are fundamental to the overall security of the network communication. How does the LoRaWAN system can make sure the communication is still running smoothly in the case of a join server being down? The answer to this question is in a backup system, which can provide the key backup and recovery.

Backward compatibility would result in a lower security grade. The LoRaWAN v1.1 supports the backward compatibility, it means a device can be connected to a network server with a lower LoRaWAN version number. However, it introduces vulnerabilities that are associated with the older versions [22,23].

## 5. Centralized Key Management Scheme for LoRaWAN

To address the above-mentioned issues, a proposed complete Centralized Key Management (CKM) scheme is outlined in the paper. This CKM scheme has an IoT infrastructure of four components, the end nodes, the network servers, the application servers and the CKMS (Centralized Key Management Server). The CKMS is a trusted agent only dealing with the whole lifecycle of the key management, i.e., the CKMS is handling the key generation/derivation, updating, backup/recovery, and key revocation. It is a trusted agency that can be players such as government, highly reputable financial institutions or registered key management centers. The application user can trust the CKMS to do all the key management work for them in a transparent way. The CKM has four sub-schemes to address the problems in LoRaWAN v1.1.

### 5.1. Key Generation Scheme (KGS)

In the LoRaWAN v1.1 that the join server is playing the role like CKMS. However, In the CKM scheme that the CKMS is playing a much more important role than the join server in the LoRaWAN, due to the fact that a much heavier key management task to the CKMS would be assigned. At first, The CKMS will have a Key Generation Scheme (KGS) to deal with key generation regarding the session keys derivation.

The KGS uses the Modified Rabbit PRNG (refer to Section 6 for details), a proposed more efficient (v1.1 uses AES, which can be considered to be computing-heavy) key generation algorithm to generate each session key based on the previous session key or the root key; which key to use for key updating depends on which rounds of the key updating has reached. In Section 6, the paper will also analyze and evaluate the significance of using the Modified Rabbit PRNG compared to the AES-ECB or the original Rabbit PRNG, since it shows a much faster computing speed and achieves a similar degree of randomness to other PRNGs, which means the energy required to generate each key is much lower. Also, it allows the key to be updated more frequently. It is essential for the LoRaWAN devices due to the majority of them are battery-based. Therefore, it would be much harder for cryptanalysts to crack the session keys, meanwhile, the lifetime of the battery in the device is extended.

### 5.2. Key Updating Scheme (KUS)

As the LoRaWAN v1.1 only recommends two periodic updating methods (1. Update in a certain period. 2. Update when a counter value is reached.) which are good, but a larger number of keys updating methods can be developed to make it more completed and secured. Hence, a complete updating scheme is detailed in this section.

**Figure 3:**
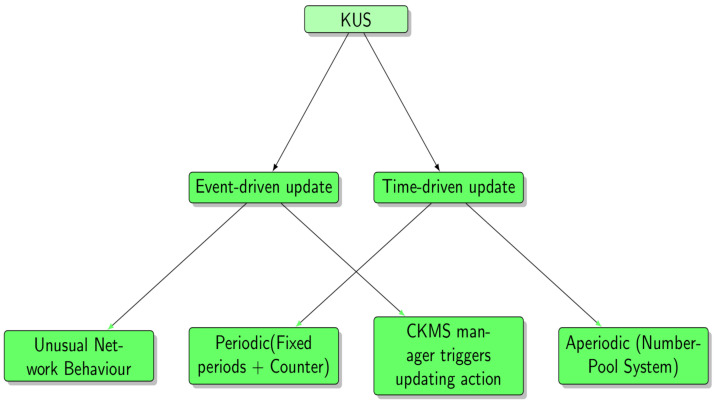
A tree-diagram illustrates KUS.

In the KUS, there are two types of triggers to update the session keys (see Figure 3). Firstly, the event-driven triggers. This type of trigger is used when a major event is happening in the network such as a large-scale network intrusion or unusual behavior has been detected in the network that would result in a key updating procedure. Hence the CKMS would use KUS to update their keys in the CKMS servers. Hence, even the attackers might acquire the keys during the event. They would still fail to decipher the contents as the results of the key updating. In this case that the CKMS can either has their own detection libraries or outsourcing the detecting role to a security specialist firm to let them inform CKMS when the network suffers or has some unusual behavior to make CKMS update their keys. On top of that, the CKMS manager can also update the keys as they desire to do so at any time, i.e., the CKMS manager can update all the keys at one single action to result in all the keys changed by using the proposed key random generator (KGS).

Secondly, the time-driven updating procedure. This procedure can be either periodic or aperiodic. For a periodic update, an end device and corresponding servers can update their paired keys at a certain period. For example, when a new pair of keys have been deployed in the field, a clock has been ticked down, a pair of new keys would be updated as the clock’s time reach its end of the period. Or the key updating would occur when both end devices and corresponding servers have communicated over a certain number of times, then the updating procedure would be triggered. On the other side, an aperiodic update means the updating procedure can happen at any time. KUS would use a number-pool system; i.e., the keys would be updated once a pre-figured number is drawn from the pool.

However, both methods need to be carefully monitored by the CKMS side. Due to the LPWAN devices usually battery-based that the users would not want their devices to drain out too soon as the result of key updating which would impact on the users’ experience. Therefore, the CKMS must ensure the key updating is balanced without occurring too much unless there is a security concern. A balanced update means that the power consumption of key update in the devices is balanced with the security requirements. For example, a battery has a 100% lifespan, then to constraint the power usage on key updating that the KUS can set up a maximum updating number for key updating. A portion of the energy would be dedicated to key updating such as a total 10% of its lifespan. Then 10 cycles of key updating might be set with each cycle of 1% of energy dispatch. Any one key update would count as one cycle no matter what types of trigger. Once it reaches its maximum number of cycles, the CKMS could decide whether to update the keys by comprising the energy. In addition to that, every end devices should be classified as their security levels, either low or high-security level. It requires a lower number of keys updating for a low-security end device. However, it requires a more frequent key updates for a high-security end device even though that might need to compromise the energy. Therefore, CKMS would decide the frequency of key updating base on their security requirements.

Furthermore, the high frequent key update does not make sense if the device is not making any communication, hence counter-based key updating should have higher priority than updating with a fixed period. A priority list for full key updating methods is recommended as follows: CKMS manager triggers key update > Unusual Network Behavior > Aperiodic (Number-Pool System) > Counter Method > Fixed Periods Update. Hence, the CKMS should follow the priority list from top to bottom in implementing the key update scheme, where ‘CKMS manager triggers key update’ has the highest priority and ‘Fixed Periods Update’ has the least priority.

### 5.3. Key Back-Up/ Recovery/ Revocation Scheme (KBURRS)

To make the LoRaWAN system running more smoothly to prevent the case of servers’ failure. A backup and recovery scheme are discussed in the section. Although in the design that the CKMS is one single entity dealing with the key management lifecycle. However, CKMS is the key management server that can be as many as it is required by the network. It only appears as one single entity from the application user’s point of view. Each CKMS is designed to cover a certain population (end devices) of the area. Hence, it depends on the recourses of CKMS party; it might require at least two CKMSs for one area that has a high density of usage (one server is for daily use, another is for backup). It also can use a shared-CKMS scheme that two close-related CKMSs can use as part of their storages to backup other’s keys. The KBURS would trigger the backup-server (another CKMS nearby) when an unwanted situation happened. Hence, all the old keys would be recovered, and all the participant parties in the network would roll back to the previous state when the unwanted situations occurred. Furthermore, when the old keys are no longer in use, it will be removed from the CKMS.

### 5.4. Backward Compatibility Scheme (BCS)

**Figure 4:**
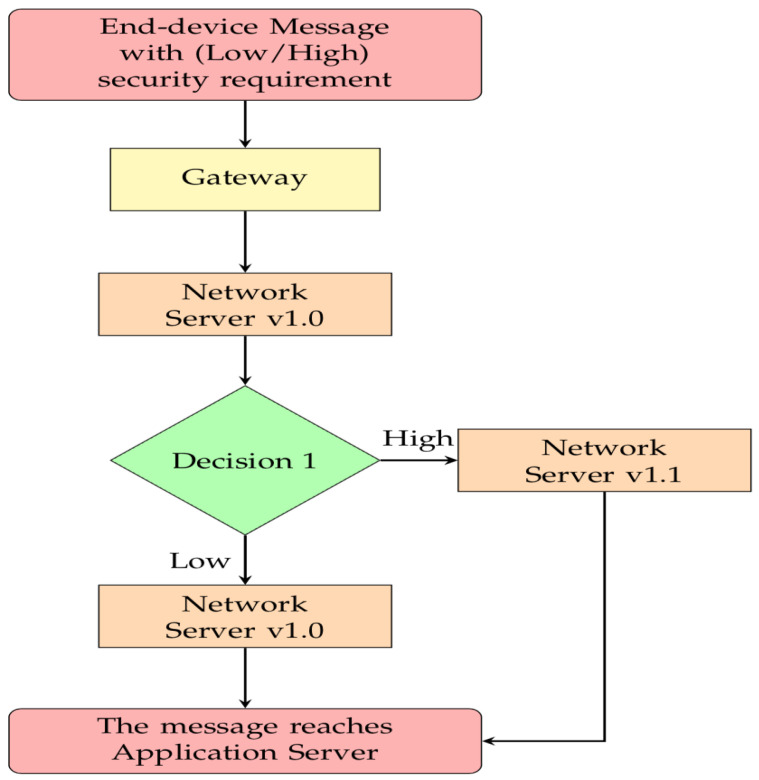
Flowchart for ensuring the Backward Compatibility in BCS.

Current LoRaWAN v1.1 supports backward compatibility. However, it would result in a downgrade in security as the security issues that happened in the lower version would emerge as the v1.1 end device tries to connect with a version number lower than v1.1 network server. Hence, this would result in a lower protocol to be used in that case. Therefore, in the BCS that the lower version network servers would tell the end device what to do based on the requirement of the device-security level, i.e., it would continue the processing for a low-security level end device. However, for a high-security level end device, the network server would redirect the traffic to a nearby v1.1 network server (See Figure 4).

## 6. New Key Generation Algorithm for LoRaWAN

Energy consumption is a key consideration for the key generation of the LoRaWAN devices due to constrained-energy supply on the devices. The proposed algorithm for key generation must satisfy the following criteria. (1): The algorithm must produce the same output key for the paired sender and receiver when the same input seed is used. In other words, if the sender and receiver use same input key on each side; then they should generate an identical output key on each side (The end device must be able to derive the same output session key which same at the network/application server). (2): Computing-efficient is needed for the algorithm. (3): The output of the key generation should be randomly distributed.

A key generation scheme can be considered to be a pseudo-random number generator. There are many existing algorithms that could be used as the generator, including the AES and Blowfish [29], another widely used block cipher. The initial test has shown that Blowfish and AES are comparable in computing efficiency for a small size input data, and this has also been verified in many published papers [30,31]. The Rabbit cipher [32] is a famous stream cipher that is well known for its speed as it is one of the algorithms in the final eSTREAM project in 2008 [33]. The reason that the Rabbit cipher is chosen from the eSTREAM project to be our proposed base algorithm due to it uses a 128 bits key that is the same as the AES 128 used in the current version of LoRaWAN. Hence, it is compatible, there is no need to change the setting of the input key size. Also, it has been proposed to use for the Wireless Sensor Networks [34]. Furthermore, unlike other final winners in the project, the Rabbit algorithm did not have the drawbacks that appeared in the other algorithms. For example, HC-128 is a table-driven algorithm, it is costly to initialize the cipher. Hence, it is not suitable for application that requires frequent key updates [35]. SOSEMANUK and Salsa20 have also been found to be vulnerable under attacks [36,37,38]. Therefore, the Rabbit cipher is a well-chosen choice for our proposed scheme.

The Rabbit algorithm is actually a pseudo-random generator and the initial test has shown that the Rabbit algorithm is faster than AES. After a further study on the Rabbit algorithm, a modified Rabbit algorithm is proposed to further speed up the computing.

The Rabbit cipher takes a 128-bit secret key with a 64-bit of IV as input, that produces an output of 128 bits keystream. The keystream then XORed with plaintext to produce the ciphertext. Here, only the keystream generation part is interested in the research. The Initial Vector (IV) in the Rabbit algorithm is for more nonlinearity and diffusion. It is important for the Rabbit as a cipher. However, as a key generator, the input key to the Rabbit algorithm will meet the basic requirement. By removing the IV and IV setups from the algorithm, the simpler Rabbit is still a decent pseudo-random generator which was introduced by Boesgaard et al. in 2003 as a cipher [39]. Hence, the proposed Modified Rabbit is based on the simpler Rabbit, but the proposed Modified Rabbit is faster than the latest Rabbit [32], please see the experimental section.

**Figure 5:**
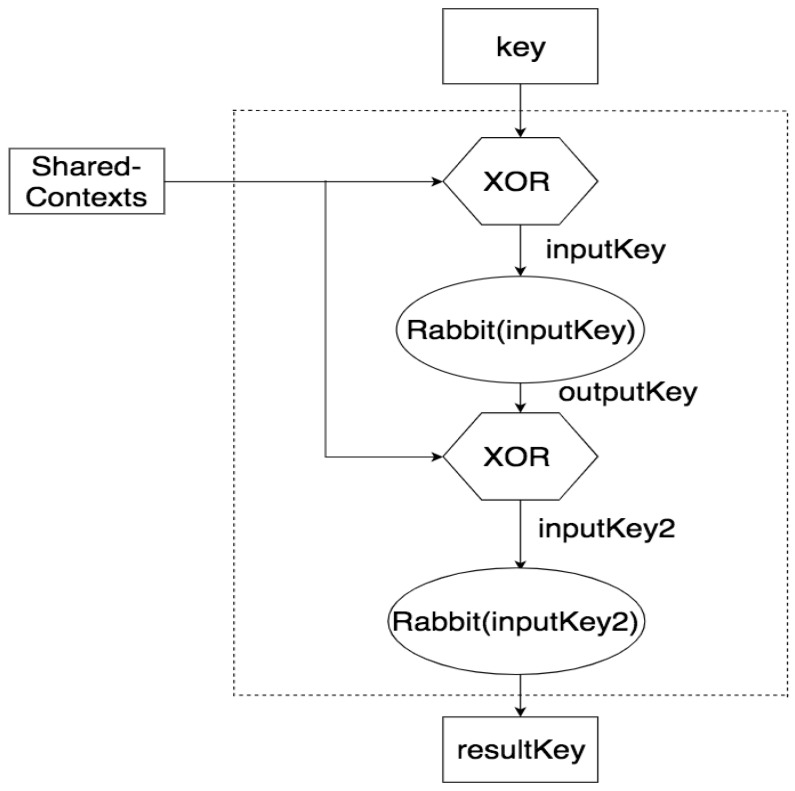
The proposed Modified Rabbit PRNG.

There are two versions of the proposed Modified Rabbit PRNG (v1 and v2). Our Modified Rabbit PRNG v1 has been published in [6]. The difference is the number of iterations in the system. Proposed Modified Rabbit has the following procedures (refer to Figure 5 for its structure): (1). XORed the key with some shared-contexts, the shared-contexts can be found on both sides of the link such as DevNounce, JoinEUI, and JoinNounce, etc. (2). The result goes through the Rabbit PRNG with two system iterations (Modified Rabbit v1) in the Rabbit PRNG (The original Rabbit has four iterations to ensure the safe margin) [39]. Or the result goes through the Rabbit PRNG with only one system iteration (Modified Rabbit v2). (3). The result from the Rabbit PRNG then XORed with the shared-contexts. (4). After that, the result once again passes through the Rabbit PRNG with the number of system iterations either two for Modified Rabbit v1 or one for Modified Rabbit v2. (5). The resulting key from the second Rabbit PRNG is the final key produced by the proposed Modified Rabbit PRNG.

Every step in the proposed PRNG is to ensure the greater key diffusion. Since the Rabbit is based on the simpler Rabbit [39] that does not contain the IV. The proposed PRNG brings 128 bits shared-contexts into the algorithm to make more nonlinearity and more diffusion into the system. Also, due to the proposed Modified Rabbit used cipher feedback with one more round that goes through the Rabbit PRNG. Hence, the result has given a considerable safe margin. The test results and analysis are conducted below.

**Figure 6:**
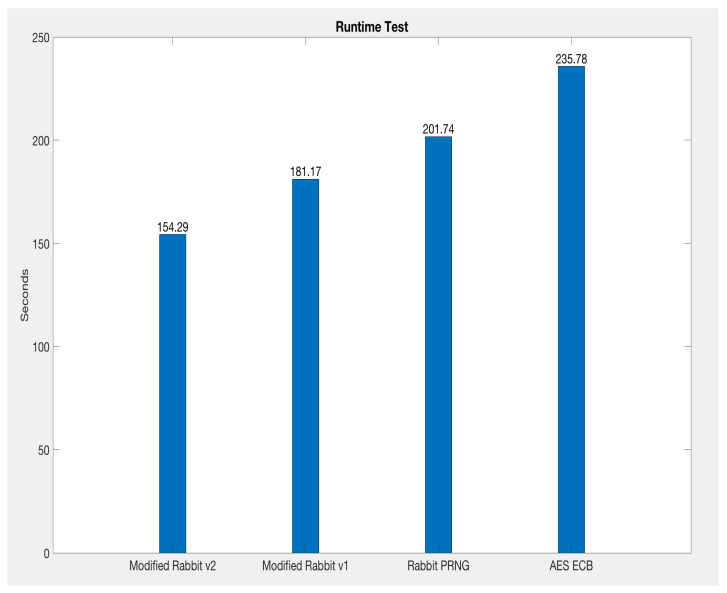
Runtime Test: Modified Rabbit v2 vs Modified Rabbit v1 vs Rabbit vs AES ECB.

A computing experiment is conducted on the Modified Rabbit PRNG v1 [6], Modified Rabbit PRNG v2, Rabbit PRNG, and AES_ECB. The test environment is a MacBook Pro with the processor 2.8 GHz Intel Core i7, 16 G.B., 1600 MHz DDR3 Memory, macOS High Sierra v10.13.6 Operating System. The 1 million repeat test is carried on each of the algorithms. The computing time comparison is shown in Figure 6. As seen from the figure, the Modified Rabbit v1 [6] is about 10.2% faster than Rabbit PRNG and 23.2% faster than AES_ECB. However, the proposed Modified Rabbit v2 is about 14.8% faster than the Modified Rabbit v1, 23.5% faster than Rabbit PRNG and 34.6% faster than AES_ECB. Hence, the proposed Modified Rabbit v2 is the most computing-efficient algorithm among the four key generators.

**Figure 7:**
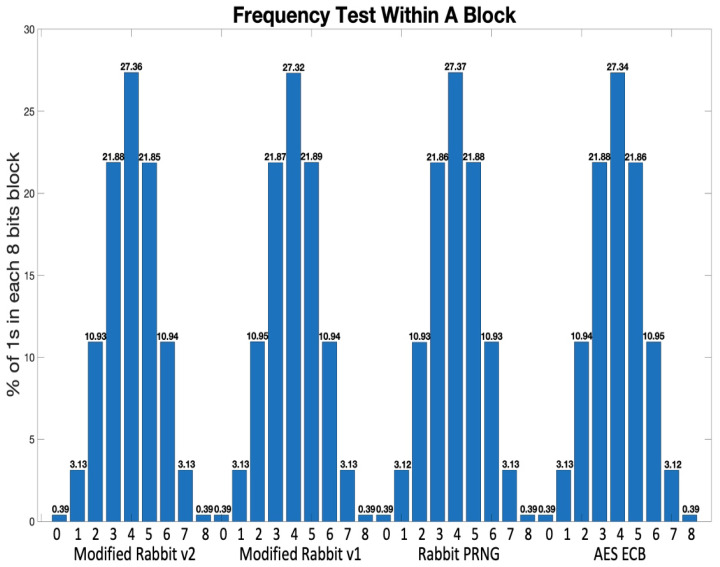
Frequency Test Within A Block: Modified Rabbit v2 vs Modified Rabbit v1 vs Rabbit vs AES ECB.

Randomness tests were also carried out on the keys generated by AES, Rabbit, the Modified Rabbit v1 and v2 algorithms. The tests follow the NIST Randomness Guidelines [26]. The first test is on the 0s and 1s distribution in the whole 128-bit key. All four algorithms have produced similar results, i.e., the percentage of 1s in the key stream is about 50%. The second test is on segments of the 128 bits. The 128 keys are partitioned into 16 segments with the segment size: 8. The number of 1s in the segments for all four algorithms follow almost the same distribution shown in Figure 7.

In addition, the Modified Rabbit v1 and v2 were also tested to verify the key uniqueness. In LoRaWAN v1.1, The session key update recommendation suggests that the session key should be updated at least every 17 minutes. Therefore, the Modified Rabbit v1 and v2 have tested in a range of 100 years. That means the proposed PRNG key generator can guarantee 100% uniqueness in the keys only if the test result shows 100% uniqueness. In the test trials, 100 years equals to 3,091,800 key updates. The results have shown that the 100% uniqueness in the keys which means that no repeats have been found in 3,091,800 keys. As the study did not do an exhaust test, the average of repeat key appearance may need far more than 3 million key generations.

## 7. Analysis of Solutions

The section provides a discussion about our proposed solutions and how our contributions are made to the field. To address the LoRaWAN v1.1 security issues listed in Section 4.5. The paper has proposed a new complete Centralized key management (CKM) scheme that included four sub-schemes.

First, the paper provides a Key Generation Scheme (KGS) that used a proposed new key generation algorithm, called Modified Rabbit v2 (Version 2) to generate the session keys. Our proposed key generation algorithm is much more efficient, 34.6% faster compared to the key generation algorithm AES_ECB 128 introduced in the LoRaWAN v1.1. It is 23.5% faster than the Rabbit PRNG. Also, the speed efficiency is about 14.8% faster than the key generation algorithm in the paper [6]. The experimental details about the Modified Rabbit v2 can be found in Section 6. From the theoretical point of view, our proposed algorithm is faster than the AES_ECB 128 is because that we do not use the computational heavy operation like the lookup table [32,40] (Substitution box, named as the S-box) in the AES. Compared to the Rabbit [32] and our earlier publication [6], our proposed algorithm used two Rabbit ciphers (Each with 1 system iteration) and two Exclusive OR (XOR) operations. Although we used two Rabbit ciphers, compared to the Rabbit (4 system iterations), and the algorithm in [6] (2 system iterations), we used less system iteration (only 1 system iteration). By using the layout in Figure 5, our proposed algorithm is much efficient compared to others, but also the randomness and uniqueness of the secret keys are ensured, details can be seen in Section 6. Hence, our KGS used such proposed key generation algorithm allows a more frequent key update to increase the difficulty of cracking the session key. Unlike the static root key updating scheme proposed by [8], we allow more frequent session key updates that make it the attacker much harder to crack the session key or even reverse-engineering the root key. At first, the attacker has more difficulty geting the current session key due to the high frequent key changes. Secondly, that makes it much harder for the attacker to reverse back the root key from the session key for the same reason. So, the need for updating the static root keys are reduced. Also, unlike [7,9,10], they used Public Key Cryptography (PKC) for the key exchanges, according to [41,42] the PKC requires more computation resources than symmetrical cryptography, and our session key generation uses the symmetrical method that can be done on both sides independently. Hence, we have less computational requirement compared to the PKC method, which is a highly important factor for the LoRaWAN constrained end devices because most end devices are battery powered. Furthermore, we used NIST Randomness Guidelines [26] to strictly ensure our keys are randomized enough, also, we conducted the uniqueness tests to make sure no repeat keys existing in our generated keys. More details can be found in the last three paragraphs of Section 6.

Second, unlike the other key management schemes described in Section 2, they are mainly focused on one aspect of the key management issues. Our paper proposed other solutions to cover the research gaps where the other papers did not cover. A table with the different gaps that the other papers and our paper have covered can be seen in Table 1. Table 1 includes the publishing year, the authors of the papers, the reference to the papers, the research gaps/questions, and the solutions to the research gaps. As seen in Table 1, our paper covered areas such as key updating mechanism (the mechanism included, when and how does the key should be updated, it has expanded the current key updating methods introduced in the LoRaWAN v1.1. More details can be found in Section 5.2), including the Key Back-Up/ Recovery/ Revocation Scheme (KBURRS) and Backward Compatibility Scheme (BCS) to handle the key during Back-Up/ Recovery/ Revocation stages, and tells the end device what to do based on the requirement of the device-security, in such guidance, the security of the keys can be further protected. By providing more key management solutions/guidance that we can ensure the keys are more secured. Hence, the security of the LoRaWAN v1.1 can be greatly protected.

In conclusion, our proposed complete key management scheme covered the areas such as: 1. The session key generation algorithm which has proved to be better than other algorithms listed in the experimental section of Section 6. 2. We provided guidance on the Key Updating Scheme (KUS), Key Back-Up/ Recovery/ Revocation Scheme (KBURRS), and Backward Compatibility Scheme (BCS) to further protect the secret keys where some of the papers in Section 2 did not cover. Therefore, our complete key management scheme can ensure the security of the LoRaWAN v1.1.

## 8. Conclusions

In this paper, LoRaWAN v1.1 Security framework was examined and its vulnerabilities were analyzed. An enhanced key management framework was proposed with improvement in several aspects. A key generation scheme based on Rabbit PRNG is proposed to replace the recommended AES algorithm for its better computing efficiency. This new scheme has been shown to have comparable randomness and uniqueness as the AES in LoRaWAN v1.1. A full key management scheme including key update, key backup, key recovery, and key revocation has been proposed to complete the existing key framework in the v1.1. The proposed key management scheme enhances the overall security of LoRaWAN and it can be included in its future release and considered in other LPWAN security frameworks. Future research can focus on developing a more advanced lightweight key generation algorithm and improving the backward compatibility scheme of the proposed version.

## Figures and Tables

**Table 1:** Comparison between different key management solutions and our proposed solutions.

Year	Authors	Reference	Gaps	Solutions
2018	Han and Wang	[8]	Lack of root key update.	High randomness static root key updating scheme.
2018	You et al.	[9]	Inefficient key exchange protocol.	A more efficient key exchange protocol based on the Elliptic Curve Diffie Hellman (ECDH).
2018	Sanchez-Iborra et al.	[7]	Inefficient key exchange protocol.	A lightweight key exchange protocol based on Ephemeral Diffie-Hellman Over COSE (EDHOC), where COSE stands for Concise binary object representation Object Signing and Encryption.
2019	Xing et al.	[10]	Lack of root key update.	A Hierarchical Deterministic (HD) wallet for key management was used by the device and server, ECDH for key exchange.
2019	Dönmez and Nigussie	[11]	Static root key issue.	Delegated key management using a master device to manage root key.
2019	Ribeiro et al.	[12]	Key management issue related to the failure of the join server.	A secure architecture for key management based on smart contracts and blockchain.
2019	Chen et al.	[6]	Slow session key generation.	A fast session key generation to enhance the speed of session key derivation, and to ensure the high key randomness.
2021	Chen et al.	This paper	Slow session key generation, limited key update mechanisms available, lack of the Key Back-Up/ Recovery/ Revocation Scheme (KBURRS) and Backward Compatibility Scheme (BCS).	A more efficient and high randomness session key generator, guidance of Key Updating Scheme (KUS), KBURRS, BCS to make the key management scheme completed.

**Table 2:** Join-Request message.

Size (Byte)	1	8	8	2	4
Join-Request	MHDR	JoinEUI	DevEUI	DevNonce	MIC

**Table 3:** Join-Accept message.

Size (Bytes)	1	3	3	4	1	1	16	4
Join-Accept Message	MHDR	JoinNonce	Home_NetID	DevAddr	DLSettings	RxDelay	CFList (Optional)	MIC

## Data Availability

Not Applicable.
